# Unusual presentation of hypocalcaemia in a peri-operative period-cause unknown

**DOI:** 10.4103/0019-5049.65363

**Published:** 2010

**Authors:** Rachna Wadhwa, Seema Kalra

**Affiliations:** Department of Anaesthesia, PGIMER and RML Hospital, Delhi, India; 1 Department of Anaesthesiology and Critical Care, IGESI Hospital, Jhilmil, Delhi, India

Sir,

A 36 year old female patient, ASA class 1, weighing 50 kgs, underwent cholecystectomy under conventional general anaesthesia- -uneventfully.

Six hours later she threw convulsions in postoperative recovery ward where she received inj. diazepam 10 mg IV and loading dose of inj. phenytoin followed by infusion. The patient was haemodynamically stable and had no signs of respiratory distress; however, she was shifted to ICU for observation. Two hours later her consciousness level was found to be poor., She was deeply sedated; however, response to deep painful stimulus was present and pupils were of normal size and normally reacting. Phenytoin infusion was stopped;, patient’s vitals were closely monitored and following investigations were done-: -complete haemogram, renal function tests, blood glucose, liver function tests, serum albumin, serum sodium, potassium, magnesium, phosphorus and calcium. Subsequently, quick portable chest X-ray and 12-lead-ECG were performed. Chest X-ray showed no abnormality and ECG revealed prolonged QT interval.

Subsequently, patient threw convulsions again, following which she developed carpo-pedal spasm (Trousseau’s sign). Hypocalcaemia[1] was suspected and 10 ml of inj. calcium gluconate 10% was given intravenously over 10 min. Arterial blood gas and electrolyte analysis revealed normal pH and gases, serum Na^+^ was 129 mEq, serum K was 2.5 mEq and serum Ca^++^ was 0.68 mmol/L, serum magnesium and phosphorus levels were normal. Inj. calcium gluconate 10%, 10 ml, was repeated in the same manner intravenously and calcium infusion[2] and potassium correction was also started simultaneously. The infusion rate of calcium was adjusted to avoid recurrent symptomatic hypocalcaemia and to maintain total serum calcium between 8 and 9 mg%.

The patient regained consciousness but was still disoriented. Her fists were tightly clenched. Calcium infusion was tapered to 1 mg/kg/hr. Subsequent investigation reports revealed all values to be normal except for serum calcium that was 7.3 mg%. At this point, she had started responding to verbal commands but she was unable to recognise her kith and kin. Following this, she developed hypotension and total serum calcium levels were found to be 6.1 mg%. Inj. calcium gluconate 10%, 10 ml, was repeated intravenously. It was followed by an infusion of inj. calcium gluconate 10% which was continued for next 12 h. Thereafter her sensorium cleared and ECG changes and serum calcium levels also reverted back to normal. Reports of the parathormone assay were also normal. The patient was discharged successfully on the fifth day with oral supplement of calcium 1 g/day.

Causes of hypocalcaemia can be many, e.g. chronic and acute renal failure, post-thyroidectomy[3] post- parathyroidectomy, primary hypoparathyroidism,[4] vitamin D deficiency, massive blood transfusion, hypoalbuminaemia, alkalosis, chemotherapy, acute pancreatitis, etc. There have been various case reports of hypocalcaemia occurring post-operatively but majority of them are following thyroid and related surgeries. In few cases, it has been reported after pre-eclampsia[5] and cardio pulmonary by pass. The anaesthesiologist must always keep the possibility of hypocalcaemia in mind whenever a patient throws convulsions in the postoperative period and check for complete serum electrolytes including serum magnesium and phosphorus levels. Hypocalcaemia may be associated with hypomagnesaemia and hyperphosphataemia and these should be corrected. The direct cause could not be determined in our case,; however, it could be respiratory alkalosis or dilutional hypocalcaemia. Perhaps the patient was chronic hypocalcaemic and minor changes in the pH made her symptomatic. Fortunately, it was diagnosed and managed successfully.
